# Mixed Adenoneuroendocrine Carcinoma Causing Colonic Intussusception

**DOI:** 10.1155/2016/7684364

**Published:** 2016-07-25

**Authors:** Marina Morais, André Costa Pinho, Ana Marques, Joanne Lopes, Alexandre Duarte, Pedro Correia da Silva, José Manuel Lopes, J. Costa Maia

**Affiliations:** ^1^Colorectal Unit, Department of Surgery, Sao João Medical Center, Faculty of Medicine, The University of Porto, 4200-319 Porto, Portugal; ^2^Department of Anatomic Pathology, Sao João Medical Center, Faculty of Medicine, The University of Porto, 4200-319 Porto, Portugal

## Abstract

Colonic intussusception is a rare cause of intestinal obstruction in adults and is caused by a malignant lesion in about 70% of cases. Early diagnosis and treatment are essential. We present a 64-year-old male patient with right colonic intussusception caused by a mixed adenoneuroendocrine carcinoma (MANEC), presenting as a giant pedunculated polyp (54 mm of largest diameter). The patient underwent right colectomy with primary anastomosis and adjuvant chemotherapy. The diagnosis of intussusception of the colon in adults is difficult because of its rarity and nonspecific clinical presentation. In this case, the cause was a rare histological type malignant tumor (MANEC).

## 1. Background

Colonic intussusception is a rare cause of intestinal obstruction in adults, with an incidence of 2-3 cases/1000000/year [1], and is usually diagnosed in the 5th-6th decades of life, with identical incidence in men and women [2]. Early diagnosis and treatment are essential because the mesentery of the involved segment is often imprisoned between layers of the overlapping intestine and its vasculature may be compromised [3]. It is caused by a malignant lesion in about 70% of cases in adults [4] and therefore attempts to reduce the intussusception are contraindicated. Due to oncological concerns, the appropriate treatment is radical resection of the involved colonic segment [5].

Here, we report a case of right colonic intussusception caused by mixed adenoneuroendocrine carcinoma (MANEC), a rare malignant tumor presenting glandular and neuroendocrine components [6, 7].

## 2. Clinical Case

We present a 64-year-old male patient with a history of chronic renal failure, radical prostatectomy for prostate adenocarcinoma, hypertension, and dyslipidemia. The patient was found to have chronic anemia and consequently he underwent upper G-I endoscopy and total colonoscopy in July 2012. A pedunculated polyp of the descending colon was detected and endoscopically resected, revealing an adenomatous polyp of tubular structure with low-grade dysplasia.

Family history was relevant for his father death at age 76 from gastric carcinoma and his sister death at age 64 from ovarian cancer.

The patient consulted with his family doctor due to a 3-month duration colicky periumbilical abdominal pain. He denied nausea, vomiting, anorexia, weight loss, or change in bowel habits. On examination he was in good general condition, without fever. The abdomen was soft and tender on deep palpation of the periumbilical region, with no palpable masses or signs of peritoneal irritation. Laboratory workup revealed microcytic normochromic anemia (hemoglobin = 11.7 mg/dL) and chronic renal failure with no further abnormalities.

**Figure 1:**
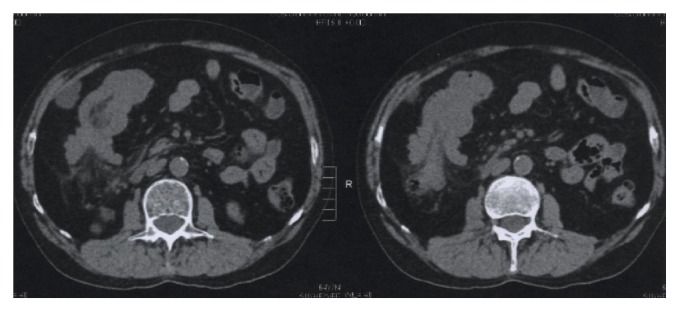
Abdominal CT (11/2012), intussusception of the right colon, proximal to the transverse colon and hepatic flexure.

As a result of pain persistence under symptomatic treatment, an abdominal CT was performed in November 2012 (Figure 1), disclosing intussusception of the right colon and no adenopathies, ascites, or liver nodules.

**Figure 2:**
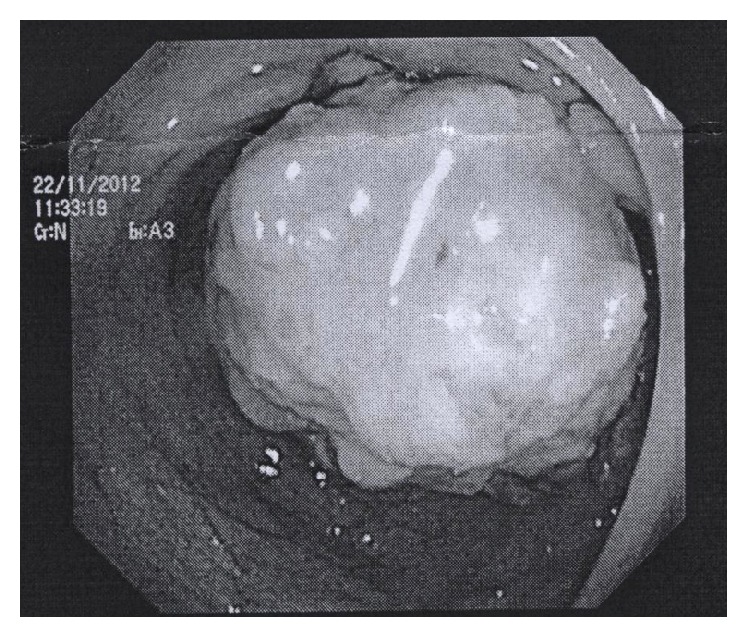
Total colonoscopy (11/2012), intussusception of the right colon by a giant pedunculated lesion.

Because of the CT findings, the patient underwent a new total colonoscopy in November 2012 (Figure 2), which confirmed intussusception of the right colon by a giant pedunculated lesion with 54 mm of largest diameter. No additional abnormalities were found in the remaining colon and rectum. Histological evaluation of biopsies performed was inconclusive due to insufficient material.

The patient was referred to our unit at this time, and the decision by the multidisciplinary team board meeting was to propose the patient for surgical treatment.

**Figure 3:**
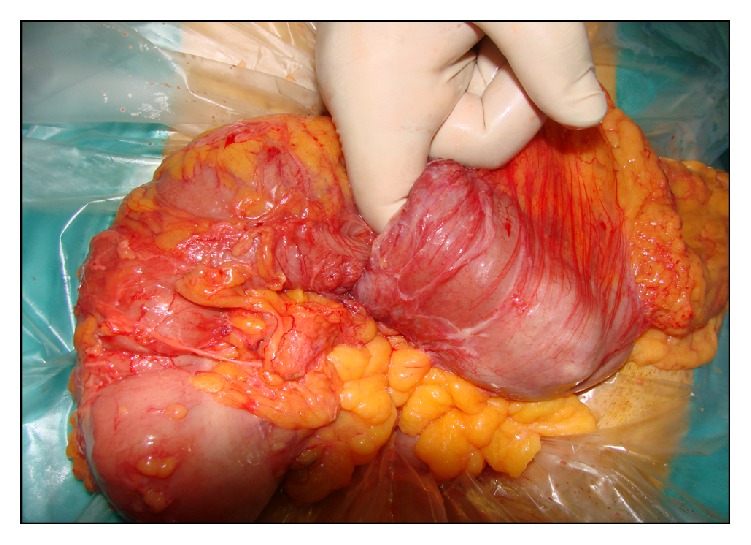
Surgery (01/2013), massive tumor of the hepatic flexure causing intussusception into the transverse colon and proximal distension.

Intraoperatively a massive tumor of the hepatic flexure of the colon causing intussusception into the transverse colon (Figure 3) and proximal distension was found. The patient underwent right colectomy with primary anastomosis.

After an uneventful postoperative period, the patient was discharged on day 6.

**Figure 4:**
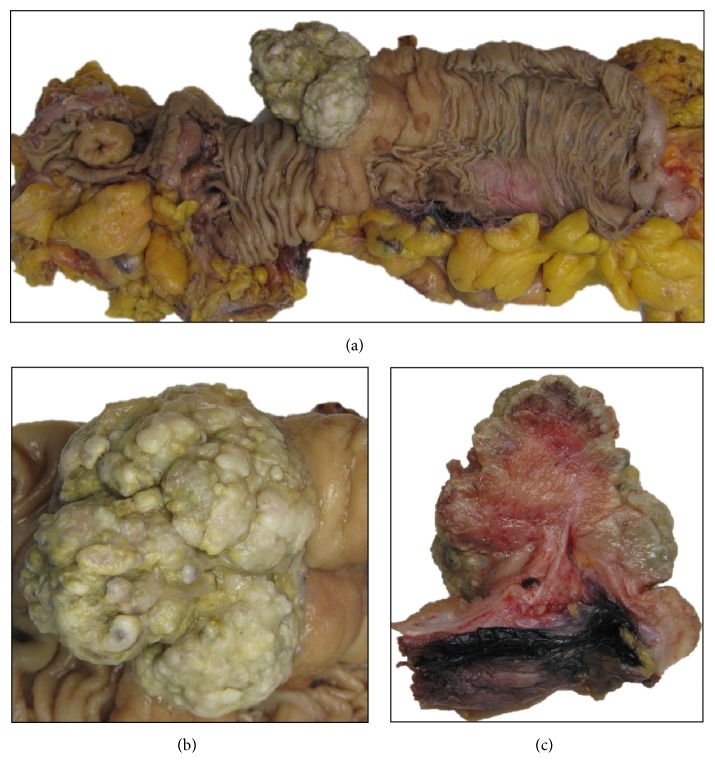
Macroscopic features of colon MANEC: surgical specimen (a) and details of tumor before (b) and after (c) section.

**Figure 5:**
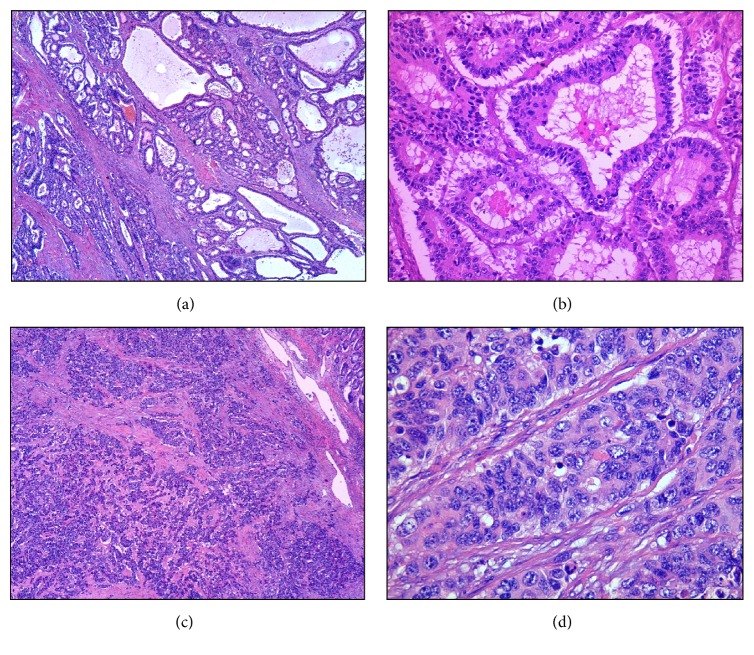
MANEC. Adenocarcinoma component ((a) HE ×40) of MANEC with tubular ((b) HE ×200) features and of NEC component ((c) HE ×40 and (d) HE ×400) with solid nests of atypical cells.

**Figure 6:**
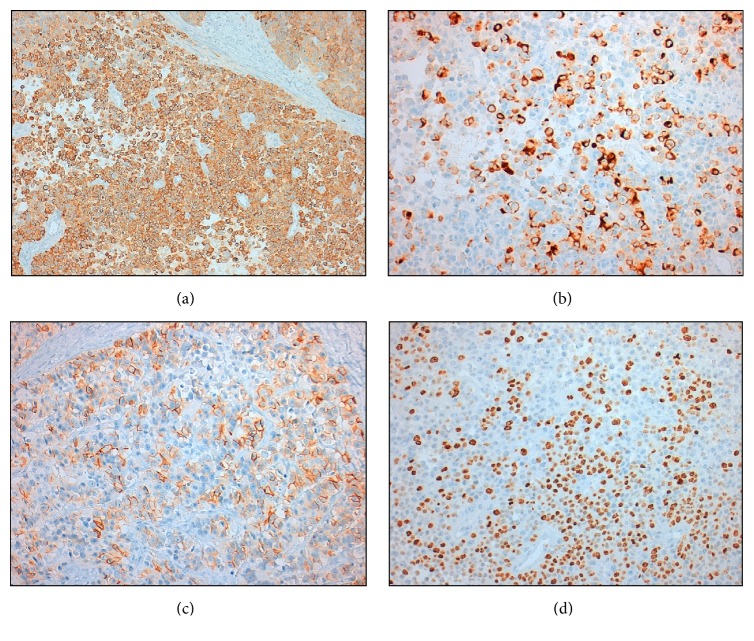
NEC component displaying diffuse synaptophysin ((a) ×100), focal chromogranin ((b) ×200) and CD56 ((c) ×200) expression, and >20% Ki-67 index ((d) ×200) in tumor cells.

Pathology evaluation of the surgical specimen revealed mixed adenoneuroendocrine carcinoma (MANEC) [8], with 30% of neuroendocrine carcinoma component (G3), invading the subserosa, metastasis in one out of 27 lymph nodes (pT3N1aR0), Dukes C, and Jass/Morson IV (Figures 4, 5, and 6).

The disease progressed, after he completed 11 cycles of adjuvant chemotherapy (FOLFOX regimen), with diffuse hepatic metastatic disease and death in June 2013. No autopsy was performed.

## 3. Discussion

Intussusception of the colon is rare in adults and is usually associated with malignancy [4]. The most common cancer is adenocarcinoma, but there are also reports of leiomyosarcomas, lymphomas, and even metastases from other malignancies [5, 9]. In the present case, a preoperative histological diagnosis was not achieved, but high suspicion of malignancy and the symptoms of the patient led to surgery [5].

The histology revealed a MANEC. MANECs are rare malignant tumors in which the glandular and neuroendocrine components coexist, with at least 30% of one of the two components. Few MANECs have been reported in the colon [6].

Due to the rarity of this entity, the best therapeutic strategy for MANECs (particularly neoadjuvant and adjuvant strategies) is not defined, and the most aggressive component should be taken into account for the decision. MANECs with well-differentiated neuroendocrine components should be treated as adenocarcinomas, while MANECs with poorly differentiated neuroendocrine components (G3) should be treated as neuroendocrine carcinomas [7].

Chemotherapy can be used for G3 neuroendocrine carcinomas (NEC) but has little role in G1 and G2 colorectal NETs [10]. National Cancer Control Network (NCCN) and European Society of Medical Oncology (ESMO) guidelines have stated that cisplatin/etoposide is the recommended chemotherapy regimen for patients with NEC [11]. For progressive disease, streptozotocin in combination with 5-fluorouracil ± doxorubicin is the most often used regimen, but the response rate is lower than 25% [10].

Concerning MANECs, adjuvant chemotherapy should also be considered, as some reports indicated effectiveness. However, due to the small number of reports, the most adequate chemotherapy regimen is still not defined [11].

The prognosis of MANECs is poor due to the frequent presentation with metastases and the absence of effective chemotherapy regimens, leading to a median survival of 7–10 months [12]. The limitations of the studies regarding MANECs are centered on the lack of information on comorbidities, heredity, and chemotherapy, which may be closely related to survival [12].

In this case report, the patient was subjected to adjuvant chemotherapy directed to the MANEC glandular component, due to lack of chemotherapy regimen directed at both glandular and neuroendocrine components, which might have an impact on survival.

The histological type of the tumor may also have implications in the follow-up. In the protocol of our unit, a colon adenocarcinoma in stage III is followed by clinical exam each 3 months for the first 2 years, each 6 months until 5 years of surveillance, and yearly after the first 5 years. The patients are evaluated with CEA determination every 3 months, abdominal ultrasound each 6 months, colonoscopy at 3 months (with visualization of the anastomotic line), yearly up to 5 years, and each 2 years after the first 5 years. The imaging studies (CT, MRI, and PET) are set aside to clarify suspicions. In the case of neuroendocrine carcinomas (WHO, G3), surveillance is performed every 4–6 months in the first year and yearly thereafter (by CT, colonoscopy, and chromogranin A) [10].

## 4. Conclusion

Colonic intussusception is rare in adults. Its clinical presentation is nonspecific which makes the diagnosis difficult. In adults, it is often associated with the presence of a malignant tumor, and its proper treatment is radical surgical resection.

MANEC is a rare histological type, which carries implications concerning treatment and prognosis. Although the most aggressive component should guide the follow-up and adjuvant treatments decisions, the best management and therapeutic strategy for MANECs remains to be defined.
